# The polyglutamine-expanded androgen receptor responsible for spinal and bulbar muscular atrophy inhibits the APC/C^Cdh1^ ubiquitin ligase complex

**DOI:** 10.1038/srep27703

**Published:** 2016-06-17

**Authors:** Laura C. Bott, Florian A. Salomons, Dragan Maric, Yuhong Liu, Diane Merry, Kenneth H. Fischbeck, Nico P. Dantuma

**Affiliations:** 1 Department of Cell and Molecular Biology, Karolinska Institutet, Stockholm 17177, Sweden; 2 National Institute of Neurological Disorders and Stroke, Neurogenetics Branch, Bethesda, MD 20892, USA; 3 Flow Cytometry Core Facility, National Institute of Neurological Disorders and Stroke, Bethesda, MD 20892, USA; 4 Department of Biochemistry and Molecular Biology, Thomas Jefferson University, Philadelphia, PA 19107, USA

## Abstract

Polyglutamine expansion in the androgen receptor (AR) causes spinal and bulbar muscular atrophy (SBMA), an X-linked neuromuscular disease that is fully manifest only in males. It has been suggested that proteins with expanded polyglutamine tracts impair ubiquitin-dependent proteolysis due to their propensity to aggregate, but recent studies indicate that the overall activity of the ubiquitin-proteasome system is preserved in SBMA models. Here we report that AR selectively interferes with the function of the ubiquitin ligase anaphase-promoting complex/cyclosome (APC/C), which, together with its substrate adaptor Cdh1, is critical for cell cycle arrest and neuronal architecture. We show that both wild-type and mutant AR physically interact with the APC/C^Cdh1^ complex in a ligand-dependent fashion without being targeted for proteasomal degradation. Inhibition of APC/C^Cdh1^ by mutant but not wild-type AR in PC12 cells results in enhanced neurite outgrowth which is typically followed by rapid neurite retraction and mitotic entry. Our data indicate a role of AR in neuronal differentiation through regulation of APC/C^Cdh1^ and suggest abnormal cell cycle reactivation as a pathogenic mechanism in SBMA.

Spinal and bulbar muscular atrophy (SBMA) is an X-linked neuromuscular disease characterized by progressive loss of motor neurons in the brain stem and spinal cord, with atrophy and weakness of bulbar and extremity muscles^1^. It is caused by expansion of a CAG trinucleotide repeat in the androgen receptor (AR) gene, which encodes a polyglutamine (polyQ) tract in the AR protein^2^. PolyQ expansions in unrelated proteins are the underlying cause of eight other neurodegenerative disorders, including Huntington’s disease, dentatorubral-pallidoluysian atrophy, and six spinocerebellar ataxias^3^. These diseases share pathological features, such as intracellular accumulation of the mutant protein in inclusion bodies^4^. Expanded polyQ tracts confer a high propensity to aggregation and impose a demand on the proteostasis machinery for correct protein folding^5^.

PolyQ toxicity is associated with alterations in ubiquitin-dependent processes, which control a wide spectrum of cellular functions, including protein degradation via the ubiquitin-proteasome system (UPS). The UPS is a major pathway for the clearance of short-lived, misfolded, and damaged proteins in both the nucleus and cytoplasm^6^. It also has critical roles in cell cycle control, signaling, and apoptosis^7^, and a general impairment of this proteolytic system could therefore provide a mechanistic explanation for the inherent cytotoxic consequences of proteins with expanded polyQ tracts^8^. It has been suggested that polyQ proteins inhibit UPS function either directly, by blocking the proteasome, or indirectly, through sequestration of essential UPS components into inclusions^9^. However, although polyQ disease proteins can cause a general impairment of the UPS when acutely overexpressed in cell lines^10^, studies in mouse models have shown that ubiquitin-dependent proteolysis is preserved in SBMA^11^ as well as other polyQ disorders^12^,^13^,^14^.

Each of the polyQ diseases has a distinct pathology with specific sets of neurons being affected^3^, indicating that cellular effects of the repeat expansion are highly dependent on the cell type and protein context. Among polyQ proteins, the physiological functions of the AR have been well characterized. AR is highly expressed in lower motor neurons in the spinal cord and brainstem^15^, a major site of toxicity in SBMA^1^, where it mediates gender differences in neural organization and neuromuscular function during development^16^. Androgen signaling remains an important mediator of axon growth and regeneration during adulthood^17^,^18^. Studies in cell and animal models have shown that toxicity in SBMA requires androgen^19^ and nuclear localization of mutant AR^20^,^21^, which is consistent with the notion that normal functions of polyQ proteins may be critical for pathogenesis^21^,^22^. While most AR functions have been attributed to its role as a transcription factor, there is also evidence for non-canonical functions of AR in cell cycle control and neurite outgrowth through direct interactions with signaling proteins and components of the cell cycle machinery^23^,^24^.

## Results

### AR-mediated neurite outgrowth is enhanced in a neuronal cell model of SBMA

**Figure 1:**
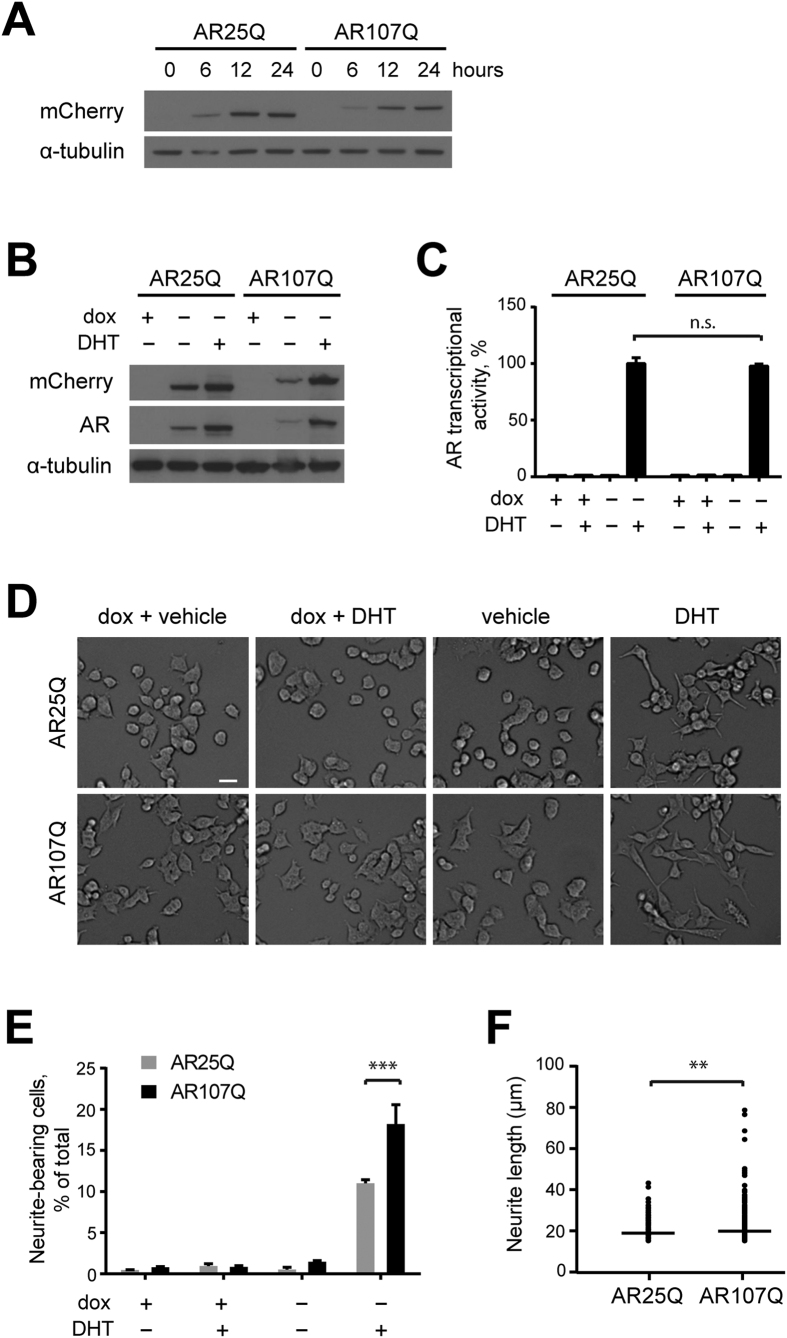
AR expression promotes androgen-dependent neurite outgrowth in PC12 cells. (**A**) Characterization of mCherry-AR protein expression in doxycycline (dox)-inducible PC12 cell lines. The cells were cultured in the absence of dox for the indicated times. (**B**) Effect of DHT on mCherry-AR protein levels. PC12 mCherry-AR lines were cultured in the presence or absence of 1 μg/ml doxycycline and 10 nM DHT for 24 hours. (**C**) Comparison of the transcriptional activity of mCherry-AR25Q and –AR107Q in the inducible PC12 cells using a luciferase reporter. The cells were incubated with 10 nM DHT 24 hours prior to the luciferase measurements. Data are expressed as mean ± SEM (n = 3). n.s. denotes non-significant differences between samples treated with DHT (Two-way ANOVA). (**D**) Representative images of PC12 mCherry-AR cells incubated in the presence or absence of 1 ug/ml dox and 10 nM DHT or ethanol vehicle for 72 hours. Scale bar, 20 μm. (**E**) Quantitative assessment of neurite outgrowth in PC12 mCherry-AR cell lines. Data are expressed as mean ± SEM (n = 3). ***P < 0.001 (Two-way ANOVA). (**F**) Quantification of neurite length in mCherry-AR-expressing cells in the presence of DHT. Each dot in the plot represents the measurement of a single cell with a horizontal line representing the median neurite length (n = 250). **P < 0.01 (Mann-Whitney test).

To study the effects of AR expression in a neuronal cell line, we generated PC12 cell lines with inducible expression of mCherry-tagged full-length human AR and normal (AR25Q) or expanded (AR107Q) polyQ tracts under the control of a tetracycline transactivator. Western blot analysis of selected clones confirmed that removal of doxycycline caused a gradual increase in mCherry-AR25Q and AR107Q protein levels, reaching a maximum after approximately 12 hours (Fig. 1A). Treatment with the androgen dihydrotestosterone (DHT) further increased protein levels of mCherry-AR25Q and AR107Q (Fig. 1B), consistent with earlier reports which showed that ligand extends the half-life of AR^25^. Cells expressing AR107Q formed nuclear inclusions that were positive for red fluorescent signal at low frequency (approximately 5%) after three days of DHT treatment (Supplementary Fig. S1). Next, we compared transactivation of a luciferase open reading frame under the control of androgen-responsive elements in these stable cell lines. We found DHT-dependent luciferase activity in AR-expressing cell lines, confirming that the mCherry-AR fusion proteins are functional in terms of ligand binding, nuclear translocation, and transcriptional activity (Fig. 1C). Since PC12 cells are devoid of endogenous AR^26^, luciferase activity was absent when transgene expression was suppressed with doxycycline. Notably, we did not detect a significant difference in luciferase activity between the cell lines expressing mCherry-AR25Q and -AR107Q, indicating that the overall transcriptional output of the two AR variants in the assay is comparable in this cell system.

PC12 cells can be differentiated in response to various soluble and genetic factors that cause cells to arrest in the G0/G1 phase and adopt a neuron-like phenotype^27^. Since AR is known to induce neurite outgrowth in neuronal cells^18^,^28^, we evaluated the effect of mCherry-AR expression on cell morphology in the PC12 cell lines. We found that both wild-type and mutant AR promote neurite outgrowth following treatment with DHT for 72 hours (Fig. 1D). This cellular phenotype was not evident after DHT treatment in the presence of doxycycline, confirming that the AR transgene was required for the androgen-dependent differentiation. Surprisingly, we found that the percentage of cells with neurites was approximately two-fold higher in DHT-treated cells expressing AR107Q compared to AR25Q (Fig. 1E). Moreover, an effect of mutant AR on neurite outgrowth was further consolidated by the finding that the average length of neurites was significantly higher in the mutant AR-expressing population compared to wild-type AR (Fig. 1F). Our findings support a role for AR in neuronal differentiation and indicate that this function of AR may be altered by the polyQ expansion.

### The polyQ expansion in AR reduces androgen-dependent cell cycle arrest

**Figure 2:**
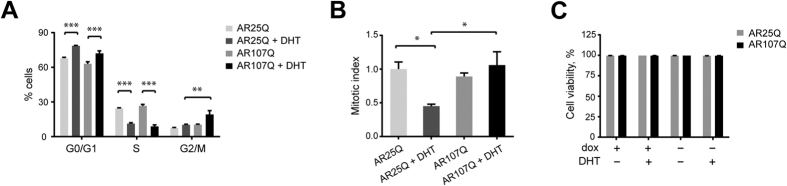
AR expression promotes androgen-dependent cell cycle arrest. (**A**) Cell cycle analysis of PC12 mCherry-AR cell lines. Cells were cultured in the presence or absence of 1 μg/ml dox and 10 nM DHT for 36 hours. Data are expressed as mean ± SEM (n = 3). **P < 0.01, ***P < 0.001 (Two-way ANOVA). (**B**) Mitotic index based on phospho-histone H3 immunostaining of mCherry-AR-expressing cells analyzed by flow cytometry. Data are expressed as mean ± SEM (n = 3). *P < 0.05 (One-way ANOVA). (**C**) Viability of PC12 cell lines following mCherry-AR expression and DHT treatment. Unfixed cells were stained with Hoechst 33342 dye and assayed for the presence of pyknotic or fragmented nuclei. Data are expressed as mean ± SEM (n = 3). No significant differences could be detected between the samples (Two-way ANOVA).

After seeing the differential effects of AR25Q and AR107Q on neurite outgrowth in PC12 cells, we next asked whether the wild-type and mutant receptors differ in their ability to induce and maintain cell cycle arrest. We found that DHT treatment significantly increased the proportion of AR25Q-expressing cells in the G0/G1 phase and significantly decreased the proportion of S phase cells (Fig. 2A, Supplementary Fig. S2), confirming previous reports that AR induces cell cycle arrest in an androgen-dependent manner^29^,^30^,^31^. A similar trend was observed with cells expressing AR107Q, however, the proportion of DHT-treated cells in the G0/G1 phase was lower compared to AR25Q and coincided with an increase in the G2/M population (Fig. 2A). To test whether the increase in the G2/M population in AR107Q-expressing cells reflects a G2 phase arrest, we analyzed the levels of phospho-histone H3, a mitotic marker^32^, in the PC12 cell lines. While the percentage of phospho-histone H3-positive cells was reduced after DHT treatment in AR25Q-expressing cells, we found that it was unchanged in cells expressing AR107Q (Fig. 2B, Supplementary Fig. S2). This finding indicates that cells did not arrest in the G2 phase but either remained mitotically active or arrested during mitosis.

Next, we tested the possibility that the altered cell cycle distribution caused by AR107Q may be a consequence of increased cell death. However, we did not detect a significant effect of mutant AR expression on viability based on nuclear morphology after staining of cells with Hoechst dye (Fig. 2C). Our data confirm that expression of the AR gives rise to DHT-induced cell cycle arrest and suggest that the polyQ expansion alters this function of AR in cell cycle regulation.

### AR physically interacts with the ubiquitin ligase APC/C^Cdh1^

**Figure 3:**
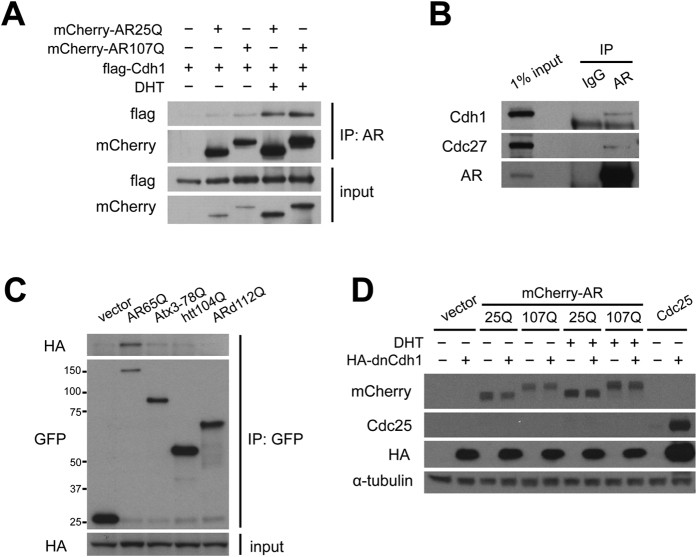
AR interacts with APC/C^Cdh1^ and is not targeted for proteasomal degradation by this complex. (**A**) Co-immunoprecipitation of Cdh1 and AR in PC12 cells. Cells were transfected with FLAG-Cdh1 and induced to express mCherry-AR. Cdh1 was co-immunoprecipitated using AR antibodies following incubation with 10 nM DHT for 36 hours. (**B**) Endogenous AR was co-immunoprecipitated from MCF7 cells after treatment with 10 nM DHT to verify association with APC/C^Cdh1^. (**C**) Co-immunoprecipitation of Cdh1 with mutant AR and other expanded polyQ proteins. HA-Cdh1 was transfected into PC12 cells together with the following GFP-tagged constructs: AR65Q, Ataxin-3 (Atx3-78Q), huntingtin exon 1 fragment (htt104Q), or an amino-terminal AR fragment (ARd112Q). Co-immunoprecipitation was performed with a polyclonal GFP antibody. (**D**) Effect of APC/C inhibition on AR protein levels. PC12 cells were co-transfected with either mCherry-AR25Q, -AR107Q, or Cdc25, and a dominant-negative form of Cdh1. After 24 hours, the cells were collected and analyzed by western blotting.

The ubiquitin ligase APC/C is a critical regulator of cell cycle progression and has important functions in postmitotic neurons. Together with its co-activator Cdh1, APC/C controls cell cycle exit and regulates axonal growth during neuronal differentiation by targeting specific proteins for ubiquitin-dependent degradation^33^. We reasoned that APC/C^Cdh1^ could be a target of AR because of the observed ligand-dependent effects of AR on both cell cycle progression and neurite outgrowth. We found that both AR25Q and AR107Q interact with Cdh1 in a DHT-dependent manner (Fig. 3A), indicating a link between APC/C^Cdh1^ activity and ligand-induced differentiation of PC12 cells. Endogenous AR in MCF7 cells also co-immunoprecipitated with endogenous Cdh1 as well as Cdc27, a core component of APC/C (Fig. 3B), demonstrating that AR and APC/C^Cdh1^ indeed form a comple*x* under physiological conditions.

The observation that wild-type AR also interacts with APC/C suggests that this binding is a normal function of this protein and not mediated by the polyQ expansion alone. Unlike AR, polyQ-expanded ataxin-3 and N-terminal huntingtin fragment did not interact with Cdh1 (Fig. 3C). Moreover, an N-terminal fragment of the mutant AR truncated 52 amino acids C-terminal of the polyQ repeat that forms insoluble inclusions in cells^34^,^35^, was insufficient for the interaction with Cdh1. These findings support the view that the association between AR and Cdh1 is not facilitated by the expanded polyQ tract alone but an inherent feature of wild-type AR.

Both wild-type and mutant AR are proteasome substrates and primarily cleared by the UPS^36^. Since APC/C has ubiquitin ligase activity, and Cdh1 is its substrate adaptor, we asked whether APC/C^Cdh1^ targets AR for proteasomal degradation. For this, we analyzed the effect of expression of an HA-tagged dominant-negative Cdh1 (DN-Cdh1), consisting of the amino-terminal 125 amino acids, on the levels of mCherry-AR25Q, -AR107Q, and a known substrate of the ubiquitin ligase complex, Cdc25^37^. While DN-Cdh1 stabilized Cdc25, it did not affect AR protein levels in the presence or absence DHT (Fig. 3D), indicating that AR is not targeted for proteasomal degradation by APC/C^Cdh1^.

### The mutant AR causes accumulation of an APC/C reporter substrate

It has previously been shown that APC/C^Cdh1^ activity is critical for cell cycle exit and neuronal differentiation through coordinated degradation of key factors that inhibit these processes in proliferating cells^38^. We therefore asked whether AR affects the function of this ubiquitin ligase complex, as differences in APC/C activity may explain the observed effects of wild-type and mutant AR on the cell cycle distribution and neurite outgrowth in our cell system. To address this question, we generated a reporter consisting of an amino-terminal fragment from *Xenopus* cyclin B fused to the green fluorescent protein (GFP). Cyclin B is a well-characterized proteasome substrate and subject to ubiquitylation by APC/C^39^. The fragment derived from cyclin B contains two destruction box (D-box) consensus motifs that are recognized by the APC/C complex. The resulting reporter construct, which we termed D-box-GFP, was stably transfected into the inducible mCherry-AR PC12 lines. We confirmed in live cells that the fluorescence of the D-box-GFP reporter gradually increases as a function of cell cycle progression and disappears during mitosis at the transition of metaphase to anaphase, which inversely correlates with the predicted APC/C activity at these stages of the cell cycle (data not shown).

**Figure 4:**
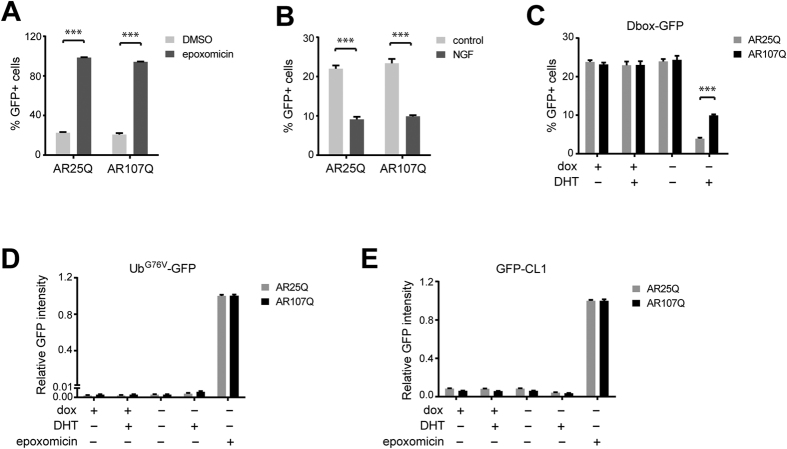
Mutant AR expression causes ligand-dependent inhibition of APC/C activity in the absence of global UPS impairment. (**A**) Effect of proteasome inhibition on the APC/C reporter Dbox-GFP in stably transfected PC12 mCherry-AR cell lines. Non-induced cells were treated with 100 nM epoxomicin for 16 hours. (**B**) Effect of neuronal differentiation on steady-state levels of the Dbox-GFP reporter. Uninduced cells were treated with nerve growth factor (NGF) for 16 hours. (**C**) Effect of wild-type or mutant AR on Dbox-GFP reporter levels. The stable PC12 cell lines were induced to express mCherry-AR25Q or -AR107Q and cultured in the presence or absence of 1 μg/ml doxycycline and 10 nM DHT for 2 days. (**D,E**) PC12 mCherry-AR cells stably transfected with the UPS reporters Ub^G76V^-GFP (**D**) or GFP-CL1 (**E**) were treated as in (**C**). As a reference, cells were treated in (**D,E**) with epoxomicin (100 nM for 16 h) to show the maximum level of reporter accumulation upon UPS impairment. Data in all graphs are expressed as mean ± SEM (n = 3). ***P < 0.001 (One or Two-way ANOVA).

In the asynchronously growing reporter lines, approximately 25% of cells had elevated levels of the D-box-GFP reporter at any given time. Treatment of the reporter cell lines with the proteasome inhibitor epoxomicin resulted in high D-box-GFP signal in almost all cells, confirming that this reporter is degraded by the UPS (Fig. 4A). In contrast, incubation with nerve growth factor caused a marked reduction in the D-box-GFP-positive cell population, consistent with a high APC/C^Cdh1^ activity associated with neuronal differentiation (Fig. 4B).

Expression of mCherry-AR reduced the percentage of D-box-GFP-positive cells in the presence of DHT, as would be expected for a condition that promotes G0/G1 arrest. Notably, the proportion of D-box-GFP-positive cells was approximately three-fold higher in the cell line expressing AR107Q compared to AR25Q (Fig. 4C). This result shows that mutant AR stabilizes an APC/C-dependent reporter substrate in a ligand-dependent manner. Increased levels of the D-box-GFP reporter may reflect changes in overall UPS functionality, which have been reported in various cellular models of polyQ toxicity^10^,^14^,^40^. Therefore, we next tested whether AR107Q affects the steady-state levels of two well-characterized UPS reporters: Ub^G76V^-GFP, which has a ubiquitin-fusion degradation signal^41^, and GFP-CL1, which contains a peptide motif targeting for ubiquitin-dependent proteolysis^10^. These proteasome substrates are recognized by distinct, APC/C-independent ubiquitylation pathways. As with the D-box-GFP reporter, these constructs were stably transfected into the inducible mCherry-AR PC12 cell lines. Levels of the Ub^G76V^-GFP reporter (Fig. 4D) and GFP-CL1 reporter (Fig. 4E) were not affected by AR25Q or AR107Q but dramatically increased upon chemical inhibition of the UPS with the proteasome inhibitor epoxomicin (Fig. 4D,E). We conclude that stabilization of the APC/C-dependent substrate by polyQ-expanded AR is not a consequence of global UPS impairment.

### Abnormal neurite outgrowth and cell cycle re-entry coincide in mutant AR-expressing cells

**Figure 5:**
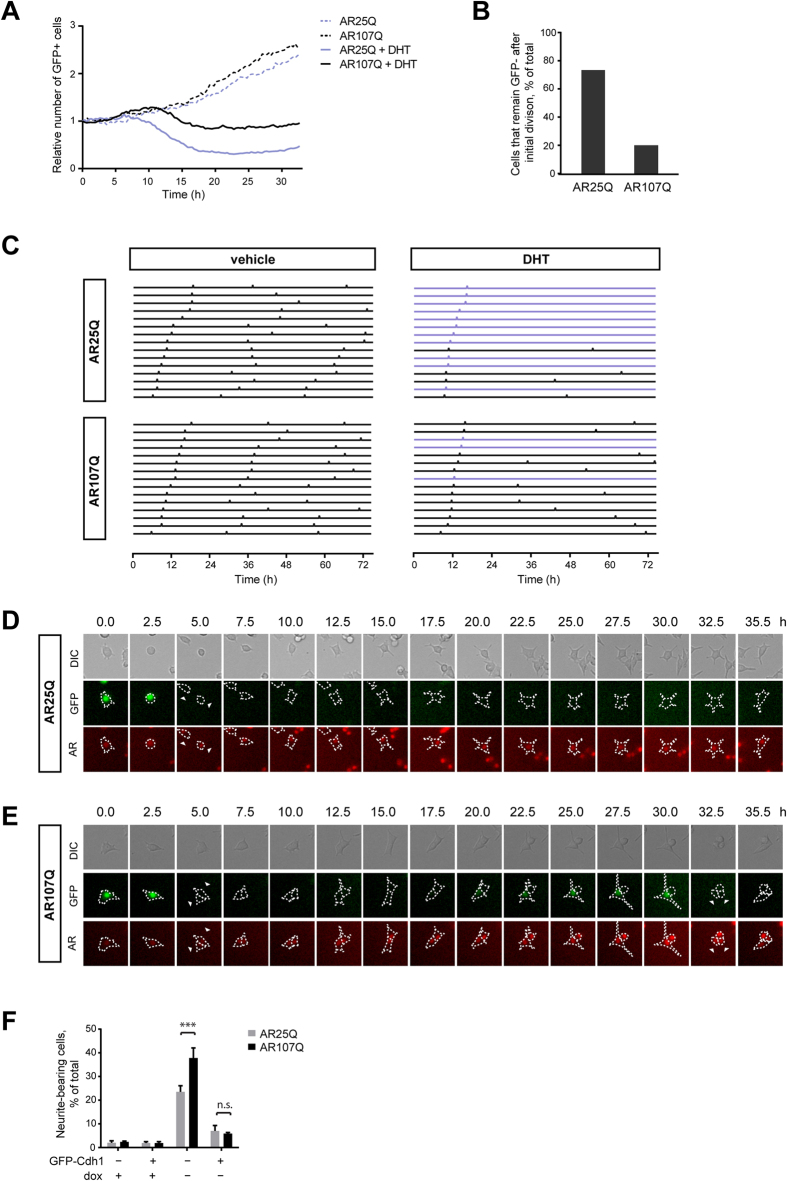
Single-cell dynamics of APC activity, neurite outgrowth, and cell division. (**A**) Time course of Dbox-GFP reporter accumulation in PC12 cells expressing mCherry-AR in the presence or absence of 10 nM DHT over 2 days. DHT was added to the cells at the 0-hour time point. (**B**) mCherry-AR expressing PC12 cells were incubated with 10 nM DHT and imaged for 72 hours. The graph shows the percentage of cells that accumulate the Dbox-GFP reporter after an initial cell division. (**C**) Dbox-GFP reporter accumulation in single mCherry-AR-expressing cells over time. Peaks indicate the maximal GFP intensity in individual cells (shown as stacked horizontal lines). (**D,E**) Representative micrographs of cells from (**C**). (**F**) PC12 mCherry-AR cells were transfected with GFP-Cdh1 or empty vector and incubated with 10 nM DHT in the presence or absence of doxycycline (dox) for 72 h. The percentage of cells with neurites was quantified by microscopy. Data are expressed as mean ± SEM (n = 3). Data are expressed as mean ± SEM (n = 3). ***P < 0.001 (Two-way ANOVA).

We performed live-cell imaging to measure the dynamics of APC/C activity in AR-expressing cells over time. PC12 cell lines expressing mCherry-AR25Q or AR107Q were imaged continuously in 10 min intervals over two days starting after the addition of DHT or vehicle, and the relative numbers of D-box-GFP-positive cells were determined as a function of time. We found a steady increase in the number of D-box-GFP-positive cells in the vehicle-treated sample during the course of the experiment, which reflects an increase in the total number of cells as a result of continuous proliferation. In the presence of DHT, the number of GFP-positive cells decreased over time relative to the value at the start of the experiment, indicating increased APC/C activity, and reached a plateau after approximately 18 hours (Fig. 5A), which is consistent with our observation of androgen-induced cell cycle arrest and differentiation of these cell lines. Importantly, the relative number of GFP-positive cells remained consistently higher in cells expressing AR107Q compared to AR25Q in the presence of DHT. The differential effect of wild-type and mutant AR on reporter levels was clearly evident within 12 hours after the addition of ligand, suggesting that altered APC/C activity may be an early event in this SBMA model.

Next, we asked whether a link exists between aberrant neurite outgrowth, cell cycle status, and APC/C inhibition in mutant AR107Q-expressing PC12 cells. Alternatively, these observations may be separate events and occur in distinct cellular populations. To address this, we monitored cell morphology as well as D-box-GFP and mCherry-AR fluorescence signal intensities in single cells over 72 hours. We found that in the presence of DHT 75% of AR25Q-expressing cells did not accumulate the D-box-GFP reporter after an initial cell division, indicating that they permanently exit the cell cycle in response to ligand. Only 26% of mutant AR-expressing cells underwent cell cycle exit after an initial division by this measure (Fig. 5B,C).

Our longitudinal analysis showed that AR25Q-expressing cells that formed neurites tended not to accumulate GFP fluorescence over the duration of the recording (Fig. 5D, Supplementary Movie 1). In contrast, cells expressing AR107Q displayed waves of elevated levels of D-box-GFP signal while developing neurites (Fig. 5E, Supplementary Movie 2). As the reporter levels reach the maximum intensity, these cells typically retracted their neurites and this event was often directly followed by cell division (Fig. 5E). Thus abnormal neurite growth occurs in the same cells that display rapid neurite retraction and mitotic re-entry consistent with the model that both events are a direct consequence of reduced APC/C activity.

Activity of the APC/C^Cdh1^ complex is regulated by phosphorylation, ubiquitylation, and association with protein inhibitors, which bind to the Cdh1 adaptor by acting as pseudo-substrates^33^. Since AR interacts with Cdh1 but does not behave as a substrate and instead reduces APC/C function, we hypothesized that mutant AR may act as such a pseudo-substrate inhibitor. If AR competes with other substrates for binding to APC/C^Cdh1^, we predicted that overexpression of Cdh1 should abrogate the cellular phenotype of the mutant protein. Indeed, overexpression of GFP-tagged Cdh1 reduced neurite outgrowth in PC12 cells expressing mCherry-AR25Q and mCherry-AR107Q (Fig. 5F). Our data suggest that this inhibitory effect of the AR is enhanced by the long polyQ tract, resulting in abortive neuronal differentiation that is followed by mitotic entry.

## Discussion

We report that mutant AR inhibits the APC/C ubiquitin ligase in the absence of global UPS impairment in neuronal cells. This effect is likely to be contingent upon native functions of AR, such as ligand binding and nuclear translocation of the receptor, as well as androgen-dependent cellular differentiation. Our data suggest that APC/C inhibition is a normal AR function that is amplified by the expanded polyQ tract. The observation that the normal AR functions are relevant for cellular toxicity^21^ also argues in favor for hypermorphic effects being responsible for the pathology as opposed to a more general gain-of-function toxicity caused by the propensity of the polyQ expanded protein to aggregate^42^. In support of this model, we found that overexpression of Cdh1 abrogated androgen-induced neurite outgrowth in cells expressing either wild-type or polyQ expanded AR. Since APC/C^Cdh1^ activity negatively regulates neurite outgrowth^43^,^44^, our observations suggest that AR inhibits the activity of the APC/C^Cdh1^ complex, a mode of action reminiscent to pseudo-substrates, which inhibit APC/C activity by competing with substrates for binding to the Cdh1 substrate adaptor^45^,^46^,^47^. It should be noted that it has been reported in an earlier study that mutant AR has a reduced capacity to induce DHT-dependent neuronal outgrowth^24^. This earlier work was performed with NSC34 cells and a possible explanation for this discrepancy may lie therefore in the different type of neuronal cell lines that have been used.

Our data suggest that the observed differences on neuronal differentiation observed with wild-type and mutant AR-expressing cells are not a direct consequence of differences in transcriptional activation. Although our molecular data support a non-translational role of AR in regulating APC/C^Cdh1^-dependent degradation, we cannot exclude the possibility that more quantitative differences in the transcription profile may contribute to this phenomenon since transcriptional activation has been assessed with a single reporter substrate. Indeed a number of studies have indicated that the polyQ repeat expansion has an effect on the transcriptional activation of AR^48^,^49^,^50^,^51^.

APC/C coordinates cell cycle transitions in proliferating cells and governs critical functions in postmitotic cells through ubiquitin-dependent degradation of a multitude of protein substrates^52^. Our observations are consistent with mutant AR-mediated inhibition of the APC/C^Cdh1^ complex since cell cycle arrest and inhibition of neurite outgrowth were simultaneously compromised in our neuronal cell model. APC/C^Cdh1^ dysregulation may also manifest in altered synaptic activity^53^ and oxidative stress, which have been reported in SBMA^54^,^55^. Although SBMA is primarily a motor neuron disease, recent studies suggest that mutant AR in the skeletal muscle may also play an important role in the pathology^56^,^57^,^58^,^59^. Abnormal stabilization of Cdh1-dependent substrates by mutant AR may also be responsible for alterations in the activity and organization of the neuromuscular junction in SBMA mouse models^60^,^61^. Given the roles of APC/C^Cdh1^ in establishing and maintaining communication between motor neurons and muscle^62^,^63^,^64^, changes in the abundance of critical substrates can therefore have both cell-autonomous as well as cell-non-autonomous effects, and may contribute to motor neuron and muscle degeneration in SBMA.

## Materials and Methods

### Plasmid constructs and cell lines

Full length human androgen receptor with 25 or 107 glutamine repeats was tagged with mCherry fluorescent protein at the amino terminus and cloned into the pTRE2hyg vector (Clontech). The APC-dependent reporter construct Dbox-GFP was generated by fusing amino acids 1–91 of *Xenopus* laevis cyclin B1 to EGFP. The Ub^G76V^-GFP reporter has been described [41] and GFP-CL1 was generated by replacing the yellow fluorescent protein in YFP-CL1 [61] with GFP.

The PC12 Tet-Off cell line (Clontech) was maintained in high-glucose Dulbecco’s Modified Eagle Medium supplemented with Glutamax, 10% heat-inactivated horse serum, and 5% fetal bovine serum at 37 °C and 5% CO_2_ (Life Technologies). The mCherry-AR constructs were transfected into PC12 cells with Lipofectamine 2000 (Life Technologies) according to manufacturer’s instructions and stable transformants were selected, isolated, and expanded in the presence of 1 μg/ml doxycycline and 100 μg/ml hygromycin. For induction of mCherry-AR, doxycycline was removed from the cells by repeated medium changes and exposed to either 10 nM DHT (Sigma-Aldrich) or ethanol vehicle in culture medium containing charcoal-treated serum (Life Technologies). Reporter cell lines were generated by stably expressing Dbox-GFP, Ub^G76V^-GFP, and GFP-CL1 in PC12 Tet-off mCherry-AR lines.

### Flow cytometry

For the cell cycle analysis, PC12 cells were pulse-labeled with 10 μM EdU for 2 hours, fixed, and stained with the Click-iT EdU Alexa Fluor 488 flow cytometry assay kit (Life Technologies) according to the manufacturer’s recommendations, phospho-histone H3 antibody (#9706, Cell Signaling Technology), and 1 ug/ml DAPI. Fluorescence intensity was assayed using the MoFlo Astrios cell sorter (Beckman Coulter) and Summit v6.2.6 software for data acquisition. Data analysis was carried out with Kaluza v1.2 software (Beckman Coulter) and standard gating methods.

### Co-immunoprecipitation and western blotting

Cells were lysed in 150 mM NaCl, 50 mM Tris-HCl pH 7.5, 1 mM EDTA, 10% glycerol, 0.2% NP-40 containing protease and phosphatase inhibitor cocktail (Thermo Scientific). Co-immunoprecipitation were performed with lysates of cells that had not been treated with DHT using indicated antibodies, followed by incubation with protein G-coupled sepharose beads (GE Healthcare). Protein samples were prepared in Laemmli buffer, separated on 4–12% Tris-glycine polyacrylamide gels and transferred onto PVDF membranes (Life Technologies). Following incubation with relevant primary antibodies, blots were visualized with peroxidase-linked secondary antibodies (R&D Systems) and chemiluminescence reagent (Perkin Elmer). The following antibodies were used: AR (sc-816 and sc-7305, Santa Cruz Biotechnology), Cdc27 (C7104, Sigma-Aldrich), Cdh1 (K0085-3, MBL), FLAG (F1804, Sigma Aldrich; 2368, Cell Signaling Technology), GFP (ab290, Abcam), mCherry antibody (polyclonal antibody raised against mRFP; kindly provided by Jacques Neefjes, Netherlands Cancer Institute), HA tag (16B12, Covance), and α-tubulin (T6199, Sigma-Aldrich).

### Luciferase assay

For AR transactivation experiments, the plasmid (ARE)_2_TATA-luc expressing firefly luciferase under the control of a minimal promoter with androgen-response elements (gift from Amilcar Flores-Morales, University of Copenhagen)^65^, and a plasmid encoding constitutively expressed Renilla luciferase (pGL4.74) were co-transfected into PC12 cells at a ratio of 10:1. Luciferase activity measurements were performed using the Dual luciferase assay kit (Promega) according to the manufacturer’s instructions.

### Microscopy

Live-cell imaging was performed on the automated image acquisition fluorescence microscope system ImageXpress (Molecular Devices) using a 20x magnification with DAPI, FITC, and Texas Red filter cubes at multiple positions. GFP reporter fluorescence was analyzed using the MetaXpress software and the multi-wavelength cell scoring application module (Molecular Devices). Long-term time-lapse imaging was performed on the fluorescence microscope system DMI6000 (Leica) at 37 °C and 5% CO_2_ using a 20x magnification with DIC, mCherry, and GFP filter sets at 4 positions per condition. Images were acquired at 10 min intervals. Images were analyzed using Volocity software (Perkin Elmer). For neurite quantification, a neurite was defined as a process extending from the soma by at least one cell diameter (10 μm). Cell viability was determined based on the relative number of pyknotic or fragmented nuclei following incubation with Hoechst 33342 dye (Life Technologies).

### Statistical analysis

Data sets were analyzed with Graphpad Prism software (version 5) for statistical comparisons. P < 0.05 was considered as statistically significant.

## Additional Information

**How to cite this article**: Bott, L. C. *et al.* The polyglutamine-expanded androgen receptor responsible for spinal and bulbar muscular atrophy inhibits the APC/C^Cdh1^ ubiquitin ligase complex. *Sci. Rep.* **6**, 27703; doi: 10.1038/srep27703 (2016).

## Supplementary Material

Supplementary Material

Supplementary Information

Supplementary Video 1

Supplementary Video 2
