# Supplementary material for: The polyglutamine-expanded androgen receptor responsible for spinal and bulbar muscular atrophy inhibits the APC/CCdh1 ubiquitin ligase complex
Source: Sci Rep. 2016 Jun 17;6:27703. doi: 10.1038/srep27703 (PMC4911547; doi:10.1038/srep27703)
Supplement: Supplementary Material [file srep27703-s1.pdf]

SUPPLEMENTARY MATERIAL

The polyglutamine-expanded androgen receptor responsible for spinal and bulbar muscular atrophy inhibits the APC/C^{Cdh1} ubiquitin ligase complex

Laura C. Bott[1,2,#], Florian A. Salomons[1], Dragan Maric[3], Yuhong Liu[4], Diane Merry[4], Kenneth H. Fischbeck[2], and Nico P. Dantuma[1,*]

[1]Department of Cell and Molecular Biology, Karolinska Institutet, 17177 Stockholm, Sweden, [2]National Institute of Neurological Disorders and Stroke, Neurogenetics Branch, Bethesda, MD 20892, USA, [3]Flow Cytometry Core Facility, National Institute of Neurological Disorders and Stroke, Bethesda, MD 20892, USA, [4]Department of Biochemistry and Molecular Biology, Thomas Jefferson University, Philadelphia, PA 19107, USA.

[#]Present address: Department of Molecular Biosciences, Rice Institute for Biomedical Research, Northwestern University, Evanston, IL 60208, USA

*Corresponding author: Nico P. Dantuma, Phone: +46-8-52487384, nico.dantuma@ki.se

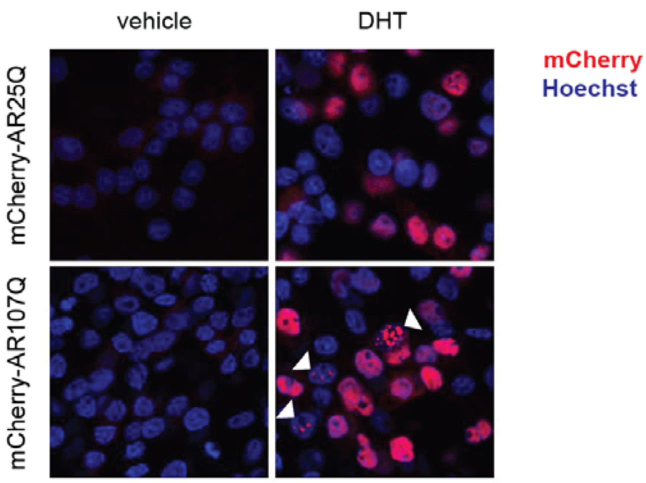

Figure S1: DHT-dependent inclusion formation in inducible PC12 cells expressing mCherry-AR107Q.
Confocal images of PC12 mCherry-AR cell lines that were cultured with or without 10 nM DHT for 4 days. White arrowheads indicate nuclei with mCherry-positive inclusions.

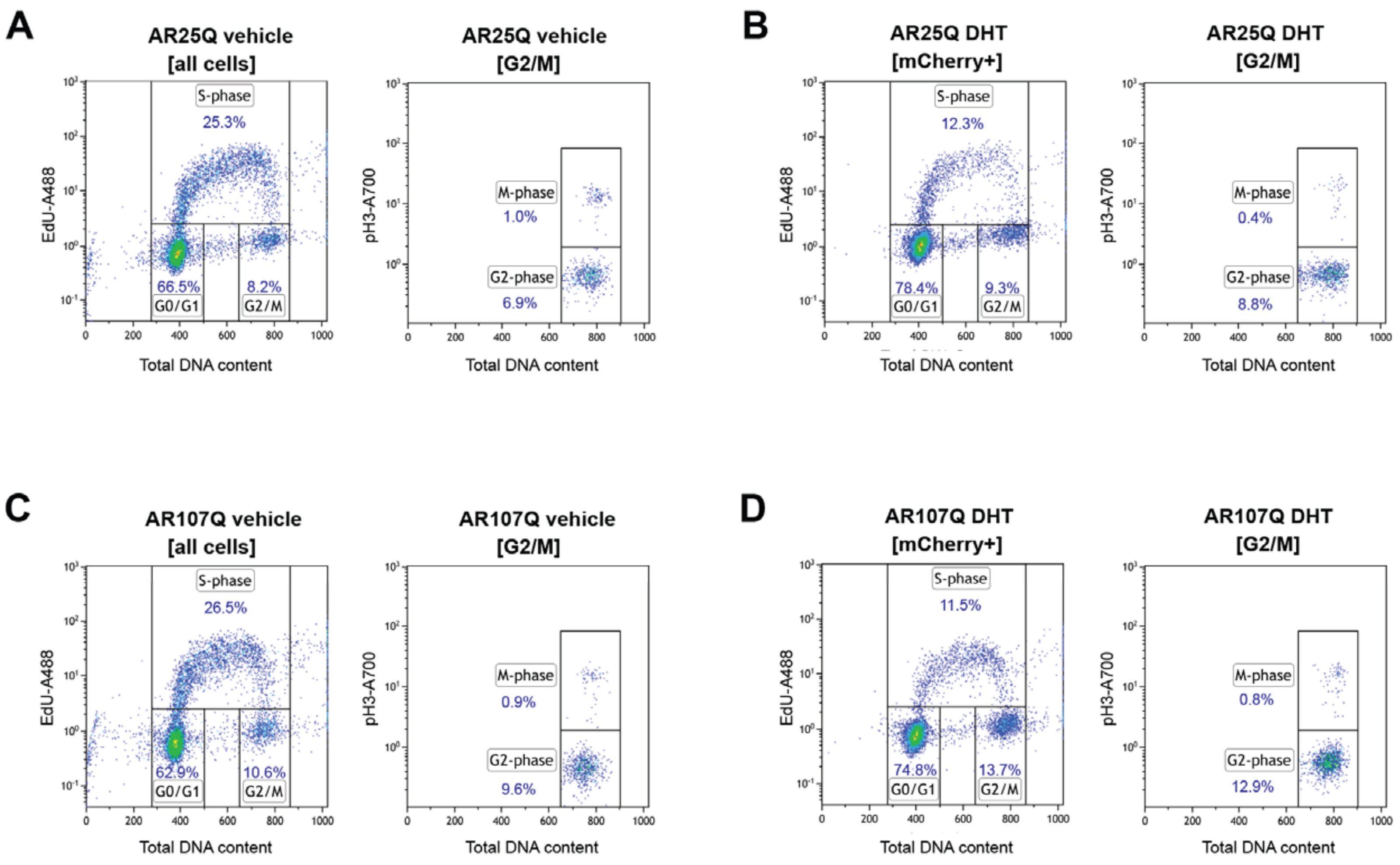

Figure S2: Cell cycle analysis of mCherry-AR-expressing PC12 cells.
(A-D) Representative FACS plots of PC12 cells expressing mCherry-AR25Q (A,B) or -AR107Q (C,D) cultured in the absence (A,C) or presence (B,D) of 10 nM DHT for 2 days. The relative distribution in G0/G1, S, G2 and M stages was determined based on fluorescence intensities of DAPI, EdU, and phospho-histone H3 stains and are expressed as percentages of the total cell population after excluding apoptotic cells.
