# Supplementary material for: The polyglutamine-expanded androgen receptor responsible for spinal and bulbar muscular atrophy inhibits the APC/CCdh1 ubiquitin ligase complex
Source: Sci Rep. 2016 Jun 17;6:27703. doi: 10.1038/srep27703 (PMC4911547; doi:10.1038/srep27703)
Supplement: Supplementary Information [file srep27703-s2.pdf]

SUPPLEMENTARY INFORMATION

The polyglutamine-expanded androgen receptor responsible for spinal and bulbar muscular atrophy inhibits the APC/C^{Cdh1} ubiquitin ligase complex

Laura C. Bott[1,2,#], Florian A. Salomons[1], Dragan Maric[3], Yuhong Liu[4], Diane Merry[4], Kenneth H. Fischbeck[2], and Nico P. Dantuma[1,*]

[1]Department of Cell and Molecular Biology, Karolinska Institutet, 17177 Stockholm, Sweden, [2]National Institute of Neurological Disorders and Stroke, Neurogenetics Branch, Bethesda, MD 20892, USA, [3]Flow Cytometry Core Facility, National Institute of Neurological Disorders and Stroke, Bethesda, MD 20892, USA, [4]Department of Biochemistry and Molecular Biology, Thomas Jefferson University, Philadelphia, PA 19107, USA.

[#]Present address: Department of Molecular Biosciences, Rice Institute for Biomedical Research, Northwestern University, Evanston, IL 60208, USA

*Corresponding author: Nico P. Dantuma, Phone: +46-8-52487384, nico.dantuma@ki.se

Supplementary information

Supplementary Video 1

Live imaging of PC12 cells expressing mCherry-AR25Q and Dbox-GFP in the presence of DHT. Still images of this movie are presented in Figure 5D.

Supplementary Video 2

Live imaging of PC12 cells expressing mCherry-AR107Q and Dbox-GFP in the presence of DHT. Still images of this movie are presented in Figure 5E.
